# The impact of chromatin modifiers on the timing of locus replication in mouse embryonic stem cells

**DOI:** 10.1186/gb-2007-8-8-r169

**Published:** 2007-08-17

**Authors:** Helle F Jørgensen, Véronique Azuara, Shannon Amoils, Mikhail Spivakov, Anna Terry, Tatyana Nesterova, Bradley S Cobb, Bernard Ramsahoye, Matthias Merkenschlager, Amanda G Fisher

**Affiliations:** 1 Lymphocyte Development Group, MRC Clinical Sciences Centre, Imperial College School of Medicine, London W12 0NN, UK; 3 Developmental Epigenetics, MRC Clinical Sciences Centre, Imperial College School of Medicine, London W12 0NN, UK; 4 Developmental Epigenetics, University of Edinburgh, Western General Hospital, Edinburgh EH4 2XR, UK; 2 Current address: Institute of Reproductive and Developmental Biology, Imperial College School of Medicine, London W12 0NN, UK

## Abstract

**Background:**

The time of locus replication during S-phase is tightly regulated and correlates with chromatin state. Embryonic stem (ES) cells have an unusual chromatin profile where many developmental regulator genes that are not yet expressed are marked by both active and repressive histone modifications. This poised or bivalent state is also characterized by locus replication in early S-phase in ES cells, while replication timing is delayed in cells with restricted developmental options.

**Results:**

Here we used a panel of mutant mouse ES cell lines lacking important chromatin modifiers to dissect the relationship between chromatin structure and replication timing. We show that temporal control of satellite DNA replication is sensitive to loss of a variety of chromatin modifiers, including Mll, Eed, Dnmt1, Suv39h1/h2 and Dicer. The replication times of many single copy loci, including a 5 Mb contiguous region surrounding the *Rex1 *gene, were retained in chromatin modifier mutant ES cells, although a subset of loci were affected.

**Conclusion:**

This analysis demonstrates the importance of chromatin modifiers for maintaining correct replication of satellite sequences in pluripotent ES cells and highlights the sensitivity of some single copy loci to the influence of chromatin modifiers. Abundant histone acetylation is shown to correlate well with early replication. Surprisingly, loss of DNA methylation or histone methylation was tolerated by many loci, suggesting that these modifications may be less influential for the timing of euchromatin replication.

## Abstract 2

A panel of mutant embryonic stem (ES) cell lines lacking important chromatin modifiers was used to dissect the relationship between chromatin structure and replication timing, revealing the importance of several chromatin modifiers for maintaining correct replication of satellite sequences in pluripotent ES cells.

## Background

DNA labeling experiments have shown that replication patterns are faithfully inherited through multiple cell divisions [1]. Individual genes replicate at similar times in each cell of a given type but locus replication timing often differs between cell types. In embryonic stem (ES) cells, the timing of DNA replication of several genes is altered in response to differentiation [2,3], which reflects changes in both gene expression and the decline in developmental potential that accompanies lineage commitment [4]. More generally, replication timing is influenced by both chromosome context [5,6] and underlying nucleotide composition [3]. Genome-wide and single gene analyses have shown that early replication timing correlates with transcriptional activity (reviewed in [7]) as well as with chromatin accessibility, or permissivity [8], and is often associated with enrichment of acetylated histones [9-11]. The exact relationship between chromatin structure and time of locus replication in S-phase remains unresolved.

Chromatin structure depends on both the action of sequence-specific DNA binding proteins and epigenetic features such as post-translational modifications of histones, the extent of DNA methylation and nuclear location (reviewed in [12]). Proteins capable of changing these parameters, chromatin modifiers, are important for establishing and maintaining particular chromatin configurations. For example, enzymes that methylate Lys4 on histone H3 or acetylate histone H3 or H4 are thought to be important for retaining accessibility whereas histone deacetylases (HDACs) and histone methyl transferases (HMTases) that target histone H3 Lys9, Lys27 and histone H4 Lys20 are important for the formation of repressive chromatin. Other factors, including DNA methyltransferases, methyl-DNA binding proteins, polycomb repressor complexes (PRCs), nucleosome remodeling complexes and Dicer-dependent short interfering RNA (siRNA), also induce or stabilize repressed chromatin states.

Recently, we showed that many genes encoding key developmental regulators replicate early in ES cells, despite being inactive at this stage [4]. Importantly, the promoters of these genes displayed an unusual chromatin profile, being enriched for both marks of active (H3K9ac, H3K4me2/3) and repressive (H3K27me3) chromatin [4,13]. This bivalent structure is interpreted as representing a 'poised' yet non-expressed state, in which H3K27 methylation is key to ensure repression [4,14,15]. Upon differentiation, many lineage inappropriate genes switch from early to late replication [2,3], suggesting that early replication of lineage specifiers in undifferentiated ES cells is actively maintained. Here, a genetic approach was used to analyze the impact of different chromatin modifiers on the replication timing profile of mouse ES cells. We show that, while early replication in ES cells correlates with peaks of increased histone acetylation, the replication times of many, but not all, single copy genes was preserved, even in mutant cells where polycomb group (PcG)-, H3K9me- or CpG methylation-mediated repression was abrogated. This conclusion is based on analysis of multiple individual genes and extended chromosome walking. The replication timing of repetitive DNA was consistently altered in many mutant ES cell lines and we demonstrate that DNA methylation is particularly important for the temporal regulation of pericentric DNA duplication in ES cells.

## Results and discussion

### Replication timing of many genes is unchanged in mutant ES cells

Mutation of chromatin modifiers *in vivo *often results in embryonic lethality and impaired development (Table 1). Despite this, murine ES cell lines lacking individual modifiers have been established and, in many cases, shown to retain multi-lineage potential. Using a panel of mutant ES cell lines (described in detail in Table 1) we examined whether a lack of specific histone methyltransferases, DNA methyltransferases, the NuRD nucleosome remodeling complex or Dicer activity was sufficient to alter the temporal profile of locus replication in ES cells. All ES cell lines examined displayed ES cell morphology, expressed markers that are characteristic of murine ES cells (such as Oct4, alkaline phosphatase and SSEA-1) and had cell cycle profiles that were comparable with wild-type ES cells (supplementary Table 1 in Additional data file 1, and supplementary Figure 1 in Additional data file 2).

**Table 1:** Characteristics of chromatin modifiers and mutant ES cells

Name	Protein function	ES cell lines	Phenotype of KO/DKO mice	Phenotype of KO/DKO ES cells	Reference
Mll	HMTase: tri-methylation of H3K4	KO: High 6	Embryonic lethal (E11.5-14.5) Homeotic transformations Mis-regulation of Hox gene expression	Mis-regultion of Hox genes Failure of *in vitro *differentiation to hematopoietic pre-cursors	[42-44]
Eed	Subunit of PRC2 Cofactor for Ezh2 (H3K27 HMTase)	KO: B1.3, G8.1	Embryonic lethal (E6.5) Failure to maintain inactive X in trophoblast derivatives	Loss of H3K27me2/3 Reduced H3K27me1 Contribute to all tissues of chimeras	[4,45-47]
Dnmt1	Maintenance DNA methyl transferase	KO: c/c	Embryonic lethal (E11.5)	Reduced DNA methylation level Reduced differentiation	[36,48,49]
Dnmt 3a/3b	*De novo *DNA methyl transferase	DKO: clone 10 (early passage)	Embryonic lethal (E11.5)	Lack *de novo *DNA methylation activity DNA methylation levels slightly reduced in early passage (severly reduced in late passage cells) Retains differentiation potential at early passages	[27,37,50]
Mbd3	Subunit of NuRD (nucleosome remodeling and HDAC complex)	KO: Fix2	Embryonic lethal (implantation)	Loss of the NuRD (nucleosome remodeling and HDAC) complex Severe differentiation block	[38,51]
G9a	HMTase: H3K9me H3K9me2 euchromatic	WT: Col4 KO: 2-3 Tg: 15-3	Embryonic lethal (E12.5)	Reduced H3K9me2 Increased H3K4me2, H3K9ac Reduced H3K9 methylation in euchromatin	[18,25]
Suv39 h1/h2	H3K9me3 (hetero-chromatic)	WT: wt26 DKO: DN57, DN72	Increased prenatal lethality Growth retarded B-cell lymphomas Male sterility Chromosome instablility in fibroblasts	Reduced H3K9me3 level; Reduced H3K9me3 at pericentric heterochromatin Increased H3K27me3 at pericentric heterochromatin Decreased CpG methylation of satellite repeats Increased transcription of major/minor satellite	[25,35,52,53]
Dicer	RNase, essential for siRNA/miRNA pathway in mammals	WT: D3 KO: D3-S5, D3-S6	Embryonic lethal (E7.5)	Increased transcription of repeats Slow growth	[30,54,55]

miRNA, microRNA.

**Figure 1:**
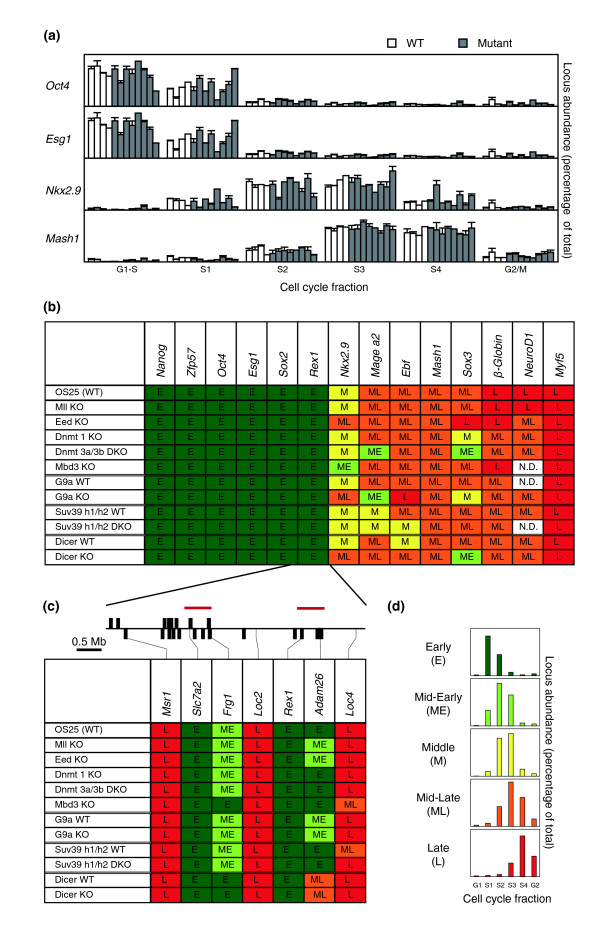
Replication timing of many genes is unchanged in ES mutants. **(a) **Replication timing analysis of *Oct4*, *Esg1*, *Nkx2.9 *and *Mash1 *in wild-type (white bars; OS25, G9a WT, Suv39 h1/h2 WT, Dicer WT) and mutant (gray bars; Mll KO, Eed KO, Dnmt 1 KO, Dnmt3a/3b DKO, Mbd3 KO, G9a KO, Suv39 h1/h2 DKO and Dicer KO) ES cells. The histograms show the relative locus replication within each cell cycle fraction as measured by qPCR for all the wild-type and mutant ES cells analyzed. The mean values and standard error of at least two independent experiments are shown. **(b,c) **Summary of replication timing of candidate genes (b) and loci surrounding the *Rex1 *gene (c). In (c), positions of genes are indicated by black boxes and the two regions changing replication timing upon ES cell differentiation are indicated by red bars. **(d) **Replication timing categories and color code. Early replication (E) is defined by peak abundance in the G1-S and/or S1 fractions, mid-early (ME) by peak replication in S2, middle (M) in S2 and S3, mid-late (ML) in S3 and late (L) replicating loci have peak abundance in S4 and/or G2.

The replication timing profiles of *Oct4*, *Esg1*, *Nkx2.9 *and *Mash1 *for four independently derived wild-type (white bars) and eight mutant ES cell lines that lack Mll, Eed, Dnmt 1, Dnmt 3a/3b, Mbd3, G9a, Suv39 h1/h2 or Dicer (gray bars) are shown in Figure 1a. Histograms indicate the abundance of newly synthesized DNA corresponding to each locus in samples prepared from sequential stages of the cell cycle (G1-S, S1, S2, S3, S4 and G2/M) for all the wild-type and mutant ES cell lines. *Oct4*, which replicates early in S-phase in all cell types analyzed, showed only minor differences between wild-type and mutant cell lines (upper panel). Similarly, there was little variation in the early replication of *Esg1 *and late replication of *Mash1 *in wild-type and mutant ES cells, even though these loci are capable of switching replication timing upon differentiation; *Esg1 *has been shown to shift to late replication upon neural induction while *Mash1 *shifts to become early replicating [2,16]. *Nkx2.9*, a neural specific gene that replicates in mid S-phase in undifferentiated ES cells showed some variation between wild-type and mutant cells. This analysis was extended to include a wider set of candidate loci that also have been shown to be permissive for changes in replication timing [2,4,17]. Figure 1b summarizes the data for 14 genes (shown in supplementary Figure 2 in Additional data file 2), in which replication timing is color-coded according to peak abundance in G1-S and/or S1 (early, green), S2 (mid-early, lime), S2 and S3 (middle, yellow), S3 (mid-late, orange), S4 and/or G2/M (late, red). Early replication of *Nanog*, *Zfp57*, *Oct4*, *Esg1*, *Sox2 *and *Rex1 *was unaffected in mutant ES cells lacking either a chromatin activator (Mll) or repressive chromatin modifiers (Eed, Dnmt1, Dnmt3a/3b, Mbd3, G9a, Suv39h1/h2 and Dicer) compared to wild-type cells (OS25 and WT). The replication of several middle- and late-replicating genes was also unchanged in chromatin modifier mutant ES cells, although three loci (*Mage a2*, *Ebf*, *Sox3*), in addition to *Nkx2.9*, showed some changes in replication patterns in the mutant lines. *Sox3 *replicated earlier in ES cells that lacked Dnmt1, Dnmt 3a/3b or Dicer but slightly later in Eed-deficient ES cells.* Mage a2 *was sensitive to loss of G9a (supplementary Figure 3 in Additional data file 2). This gene is transcriptionally regulated by G9a [18] (supplementary Figure 2 in Additional data file 2), and belongs to the *Mage *genes that are DNA methylated in adult somatic tissues [19]. Replication of *Ebf*, a gene that replicates earlier in pro- and pre-B cells than in ES cells [17], showed slight shifts in replication in G9a, Suv39 h1/2 and Dicer-deficient ES cells. From a total of 14 loci analyzed in Figure 1b, four showed a temporal shift in one or more mutant ES cell lines. We analyzed the sequence context of the genes (supplementary Table 2 in Additional data file 1) but neither GC content nor Line density was obviously different between genes that change replication timing or those that remain unchanged in the mutant cells. Bivalent genes were represented among loci that showed shifts in response to loss of chromatin modifiers (such as *Nkx2.9 *and *Msx1*) as well as those that did not change their timing of replication (such as *Math1 *and *Sox1*; supplementary Figure 2 in Additional data file 2). Some replication timing changes were in the predicted direction (that is, an advance upon loss of a repressive chromatin modifier) whereas others were counter-intuitive, which we cannot explain. Importantly, however, we did not observe consistent shifts towards earlier or later replication in response to removal of a specific chromatin modifier. These results suggest that while some loci may be more sensitive to chromatin changes than others, none of the chromatin modifiers studied here is capable of overt de-regulation of the temporal order of gene replication in ES cells. This was true even for cells lacking Eed (and hence devoid of methylated H3K27), a factor previously shown to be important for transcriptional repression and chromatin bivalency in ES cells [15]. Thus, our data do not support a model where methylation of specific histone residues or CpG dinucleotides confers replication at a certain time in S-phase. To explore this further, we also analyzed a large contiguous region surrounding *Rex1 *(Figure 1c), a gene that is expressed and early replicating in ES cells, but switches to late replication in differentiated cells and concomitantly looses histone acetylation as the gene is silenced [2]. Chromosome walking has previously identified two domains within this 5 Mb region that replicate early in ES cells and switch to late replication upon differentiation (marked by red lines in Figure 1c) [2] (P Perry and VA, unpublished). Analysis of this entire region in each of the chromatin modifier mutants showed that the boundaries of early and late replication were retained.

An explanation for why the replication times of several loci are unchanged in mutant ES cells might be that other modifications compensate for this loss - for example, increased DNA methylation might compensate for loss of Eed-mediated repression. To address this possibility we knocked-down Eed (using short hairpin RNA) in ES cells that already lacked Dnmt1 (supplementary Figure 4a in Additional data file 2) but were unable to detect additional changes in the replication profiles of early (*Oct4*, *Rex1*), middle (*Nkx2.9*) or later replicating loci (*Sox3*, *Mash1*, *β-Globin*) (supplementary Figure 4b in Additional data file 2). Collectively, these data suggest that only a minority of loci (5/23; supplementary Figure 2 in Additional data file 2) change their replication timing in response to severe reduction of DNA methylation (Dnmt 1 knock out (KO)), methylation of H3K27 (Eed KO), euchromatic H3K9 methylation (G9a KO) or NuRD activity (Mbd3 KO), despite being sensitive to changes that occur during normal differentiation [2,4,16].

### Histone acetylation and replication timing in ES cells

**Figure 2:**
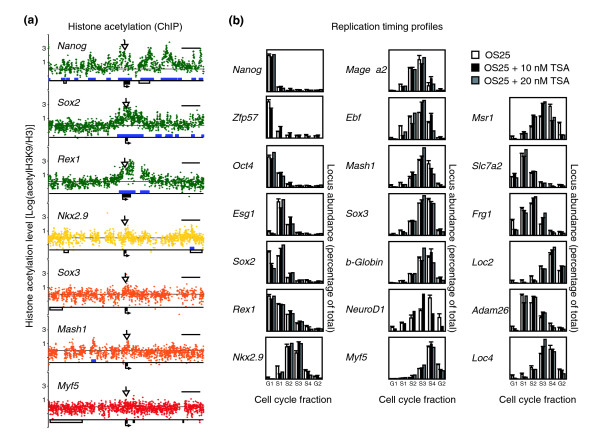
Histone acetylation and replication timing in ES cells. **(a) **The level of acetyl-H3K9 relative to total H3 is shown for each probe in >200 kb regions surrounding the candidate loci (values represent log2{acetylH3K9/H3}). The peaks of histone acetylation were identified using the hidden Markov model and are marked in blue. The location of candidate genes are shown (black box) relative to other genes (white box) within each region. Arrows show the position of the primers used for the replication timing analysis and the size bars (black) represent 25 kb. Raw data are available in Additional data file 3. **(b) **The replication timing of candidate loci in untreated ES cells (white bars) and after incubation with 10 nM (black bars) or 20 nM (gray bars) TSA is shown as histograms. The mean values and standard error from at least two (two to three) independent experiments are shown.

To assess whether histone acetylation levels are indicative of early replicating regions in ES cells, as has been suggested for other cell types [9,11], we compared the abundance of histone acetylation at the candidate loci using the chromatin-immunoprecipitation (ChIP) assay. Replication timing domains are very large (0.2-2 Mb) compared to promoter regions that are conventionally analyzed by ChIP. We therefore applied custom-made tiling arrays to examine approximately 200 kb regions surrounding the loci for enrichment of acetyl-H3K9. Early replicating loci, such as *Sox2*, *Nanog *and *Rex1*, contained numerous peaks of acetylation (eight- to ten-fold enrichment relative to H3; Figure 2a and Additonal data file 3). Loci that replicated in the second half of S-phase showed much fewer peaks and the enrichment was less pronounced (one- to two-fold). Basal histone acetylation levels were, however, relatively constant across each of the regions analyzed, irrespective of whether they replicated early or late.

To assess whether enhanced histone acetylation was sufficient to determine early replication, we treated ES cells for 24-48 h with doses of the HDAC inhibitor Trichostatin A (TSA), which raised the global levels of histone acetylation in nuclei (as judged by immunofluorescence; data not shown) without compromising cell viability, proliferation or morphology. TSA treatment of wild-type OS25 ES cells did not affect the replication timing of any of the loci tested, including the region surrounding *Rex1 *(Figure 2b). Similar treatment has been reported to advance replication of the cystic fibrosis transmembrane conductance (CFTR) gene in cell lines [20]. The failure of TSA treatment to impact on replication of these genes in ES cells suggests that either temporal shifts are highly gene specific or that HDAC inhibition by TSA treatment merely increases histone acetylation at sites that are already acetylated and early replicating in ES cells. Consistent with the latter explanation, TSA treatment was recently shown to increase histone acetylation and expression of genes such as *Hox B1 *and *Brachyury *that replicate early in ES cells (L Mazzarella and HFJ, unpublished) [21].

### Altered replication of satellite sequences in ES cells lacking specific chromatin modifiers

**Figure 3:**
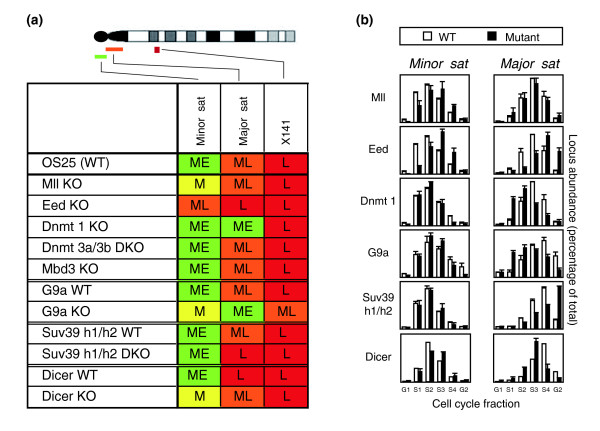
Satellite replication in ES cells is altered by mutation of chromatin modifiers. **(a) **Summary of replication timing of repeat sequences in mutant ES cell lines. Top, ideogram of the acrocentric mouse chromosome X, showing the position of minor satellite (Minor sat), major satellite (Major sat) and the X-linked X141 repeat. **(b) **Examples show replication timing of repeats in wild type (WT, white bars (OS25 for Mll, Eed and Dnmt1; matched wild-type lines for G9a, Suv39 h1/h2 and Dicer)) compared to ES cells mutant for the indicated chromatin modifier (black bars). The mean values and standard error of at least two (two to five) independent experiments are shown.

Next we assessed the replication of three different murine repeat sequences. X141 is a complex X-linked repeat that is constitutively late replicating and heterochromatic [6,22]. Minor and major satellites are simple direct repeats located around the centromeres of mouse chromosomes that, in wild-type ES cells, replicate in mid-early and mid-late stages of S-phase, respectively (Figure 3a). In mutant ES cells, late replication of X141 was retained but the timing of both minor and major satellites was altered. Minor satellite replication was selectively delayed in ES cells lacking Mll, which catalyses methylation of H3K4, an activating histone mark. The replication of both satellite sequences was delayed in Eed deficient ES cells, which lack repressive H3K27me3 (Figure 3a). Retarded replication of the major satellite was also seen in cells lacking the Suv39h1/h2 HMTases compared with matched wild-type controls. In contrast, major satellite replication was advanced in Dnmt1 KO and G9a KO ES cells. Interestingly, a comparison of matched mutant and wild-type ES cells showed advanced replication of major satellite sequences in the absence of Dicer, consistent with the proposed role of siRNA in silencing repetitive elements [23]. In a recent study, an advance in the replication of the major satellite in Suv39h1/h2 double knockout (DKO) relative to wild-type fibroblasts was reported [24], although the authors noted that this advance was, in fact, not statistically significant. The apparent discrepancy between their observation and ours could be the result of intrinsic differences in the mutant cell lines used, or reflect secondary adaptations to loss of chromatin components. In this regard, compensatory chromatin modifications have been previously described, including an increase in H3K27me3 levels in Suv39h1/h2 deficient ES cells [25].

Minor and major satellites both carry DNA methylation and share some histone marks [26], but their chromatin structure is remarkably dissimilar. Major satellite DNA replicates in mid to late S-phase in ES and somatic cells and is characterized by hypoacetylation, trimethylation of H3K9 and H4K20 and DNA methylation [25,27,28]. The minor satellite contains the centromeric H3 variant CenpA, lacks appreciable amounts of the repressive H3K9me3 and does not bind HP1 [28,29]. Instead, this repeat carries the permissive H3K4me2 mark [28] and it replicates in the first half of S-phase (Figure 3). We show that the replication timing of major and minor satellites responds very differently to mutation of Mll, Dnmt1, Suv39h1/h2, Dicer and G9a, supporting the view that the two repeats are regulated differently.

Earlier replication of the major satellite in Dicer KO cells might reflect increased repeat RNA accumulation, as has been reported in some cells upon loss of Dicer [23,30], prompting us to measure transcript levels of the repeats in each of the mutant cell lines (Table 2). Despite variation among different lines of wild-type ES cells (major 0.7-4, minor 0.1-5), a significant increase in major satellite transcript levels was seen in Dicer KO cells (17 compared with 4 in matched wild-type cells). Increased minor and major satellite transcripts were also seen in Eed deficient ES cells but not in other mutant lines that also change satellite replication timing (for example, Dnmt 1 KO). These data suggest that while chromatin modifiers can influence satellite transcript levels, precocious replication is not an invariable consequence of satellite transcription.

**Table 2:** Relative transcript levels* of repeats in ES cell lines

	Minor satellite	Major satellite	X141
WT (OS25)	0.4 ± 0.1	1.8 ± 0.5	1.8 ± 0.3
Eed KO	7.2 ± 6.5^†^	18.5 ± 17.0^†^	1.8 ± 0.2
Dnmt1 KO	0.3 ± 0.1	2.1 ± 0.0	2.0 ± 1.3
Dnmt3a/3b DKO	0.1 ± 0.0	5.7 ± 2.4	1.5 ± 0.9
G9a WT	0.1 ± 0.0	0.7 ± 0.1	0.5 ± 0.7
G9a KO	0.4 ± 0.4	1.0 ± 0.2	ND
Suv39 h1/h2 WT	4.9 ± 0.8	2.5 ± 1. 6	1.1 ± 0.3
Suv39 h1/h2 DKO	4.0 ± 1.3	2.2 ± 0.7	2.1 ± 0.5
Dicer WT	0.2 ± 0.0	4.1 ± 0.0	2.7 ± 0.0
Dicer KO	0.9 ± 0.6	17.4 ± 5.5	4.1 ± 1.2
C2C12 dif	100	100	100

*Levels of repeat sequence RNA were normalized to a house keeping gene (*Ube*) and expressed as a percentage of the levels detected in differentiated mouse myocytes derived from C2C12 (C2C12 dif), samples in which high levels of major and minor satellite transcripts have previously been detected [56]. Controls without reverse transcriptase were analyzed in parallel to dismiss contamination with genomic DNA.

^†^Four independent samples showed variable but increased transcript levels: major satellite, 8.2%, 8.7%, 13.4%, and 43.8% of C2C12; minor satellite, 2.6%, 7.1%, 11.8%, and 13.8% of C2C12. ND, none detected.

**Figure 4:**
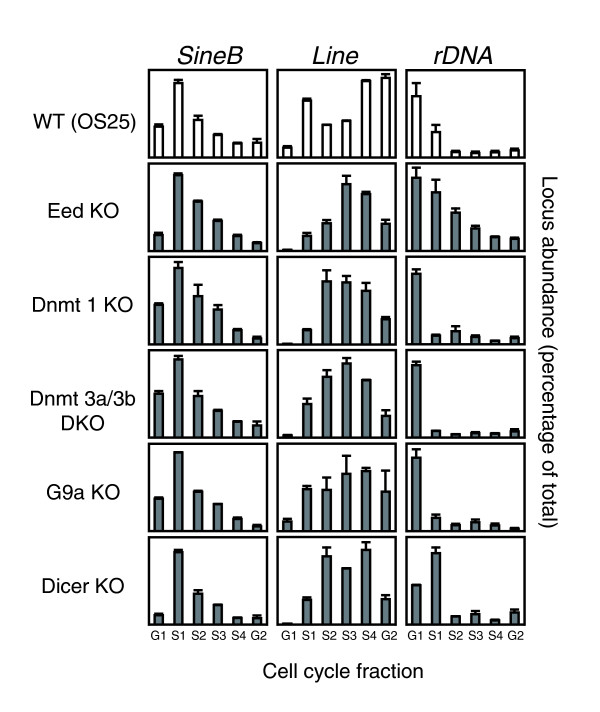
Replication timing of repetitive elements in wild-type and mutant ES cell lines. The replication timing was determined for retrotransposons (LINE and SINE B1) and rDNA repeats in wild-type OS25 ES cells and in mutant ES cells lacking Eed, Dnmt 1, Dnmt 3a/3b, G9a or Dicer. The mean values and standard error of at least two independent experiments are shown.

To determine whether satellite sequences are particularly sensitive to loss of chromatin modifiers or if this is a general repeat-associated feature, we analyzed long interspersed nuclear elements (LINEs) and short interspersed nuclear elements (SINEs), which are found as single copies interspersed with genes and other unique sequences at many locations in the genome, as well as the tandemly repeated rDNA array. In wild-type ES cells, SINE B1 replicates early whereas LINE 1 elements show replication in all fractions of the S-phase (Figure 4), consistent with the known genomic distribution of these repeats; SINEs are primarily associated with gene rich regions (which replicate early), whereas LINEs are enriched in AT-rich, gene poor regions (which often replicate late, but can change replication timing depending on the cell type [3]). The rDNA sequence, which in fibroblasts comprises an early replicating active and a late replicating silent fraction [31], replicated synchronous very early in S-phase in wild-type ES cells (Figure 4), possibly reflecting a high demand for biosynthesis in these rapidly dividing cells. Analysis of these three repeat sequences in the mutant ES cell lines revealed only very small changes with respect to wild-type cells. Replication of rDNA was extended in Eed deficient cells and slightly delayed in ES cells lacking Dicer.

### DNA methylation selectively affects major satellite replication timing

**Figure 5:**
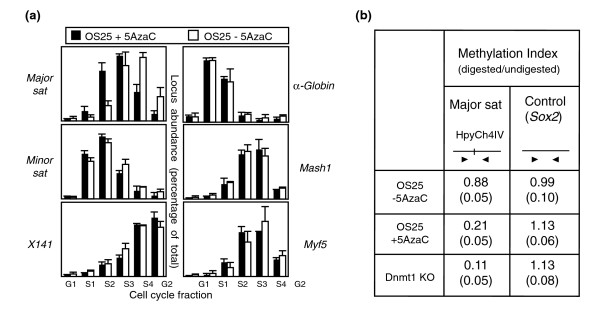
Major satellite replication is specifically advanced by 5-azacytidine treatment. **(a) **Replication timing of 5-azacytidine treated (black bars) and untreated ES cells (white bars). The mean values and standard error of at two independent experiments are shown. **(b) **Demethylation index (HpyCh4IV digested to undigested genomic DNA) of the major satellite in untreated (OS25), 5-azacytidine-treated and Dnmt1 KO ES cells. *Sox2 *(no HpyCh4IV site) serves as an internal control. Diagrams show the positions of primers and HpyChIV site. The standard deviation is shown in brackets.

Our data show that loss of Dnmt1 in ES cells (which causes genome-wide loss of CpG methylation; Table 1) results in early replication of the pericentric major satellite sequence without widespread changes in the replication timing of euchromatic loci or other repeat elements (Figures 3 and 4). To verify that reduction in DNA methylation is sufficient to precipitate this advance in major satellite replication, we experimentally demethylated wild-type ES cells. Exposure for three days to the Dnmt inhibitor 5-azacytidine reduced DNA methylation (from 0.88 in untreated to 0.21 in 5-azacytidine-treated cells, compared to 0.11 in the Dnmt1 KO ES cell line; Figure 5b) and caused an advanced replication of the major satellite (Figure 5a). The replication timing of the minor satellite as well as single copy genes (*α-Globin*, *Mash1 *and *Myf5*) was unaffected in treated cells. Collectively, these results suggest that DNA methylation *per se *is important for maintaining the correct temporal replication of the major satellite.

A role for DNA methylation in replication of heterochromatic foci has been previously observed in fibroblasts and during development [32]. Here we show that DNA methylation is important in maintaining late replication specifically of major satellite repeats in undifferentiated ES cells. As DNA methylation of the major satellite is also reduced in Suv39h1/h2 DKO ES cells (Table 1), it is perhaps surprising that these mutant cells have delayed major satellite replication (Figure 3). It is possible that other chromatin modifications compensate for the loss of H3K9me3 to ensure heterochromatin formation in Suv39h1/h2 deficient cells, an idea that is consistent with enhanced H3K27me3 at pericentric regions in these cells [25].

## Conclusion

We show that the timing of mouse satellite replication is altered in ES cells lacking specific repressive chromatin modifiers. In particular, replication was advanced by mutation of Dnmt1, G9a or Dicer, consistent with their repressive nature. Earlier replication of major satellite was also induced by 5-azacytidine treatment, demonstrating the importance of DNA methylation for correct timing of this sequence. The sensitivity of satellite repeats to chromatin modifiers may be a reflection of their complexity and size. Genome-wide studies have shown that replication timing of non-repetitive sequences is constant over large 0.2-2 Mb regions [7], which often include multiple loci that are regulated by different mechanisms. Repetitive regions, on the other hand, have a more uniform chromatin structure, which may make them more vulnerable to loss of specific chromatin modifiers. Consistent with this idea, the major and minor satellites comprise simple direct repeats with high copy numbers (50-200,000) [26] whereas the stable X141 is part of a much more complex repetitive region and is represented only 80-90 times in the mouse genome [22]. Interestingly, the size of the late replicating fraction of the tandemly repeated rDNA array in fibroblasts was shown to depend on NoRC, an ATP-dependant chromatin remodeling complex [33].

Of the single copy genes examined in the study, we show that the replication timing of some loci are more sensitive to the loss of individual chromatin modifiers than others. Overall, the apparent stability of gene replication profiles in mutant ES cell lines suggests that for many single copy loci, replication timing is not primarily controlled by methylation of specific histone residues or DNA methylation, but, in agreement with previous studies [4,8,9,11], histone acetylation is shown to be a good predictor of replication timing. These data are consistent with a mechanistic link between early origin firing and acetylation in mammalian cells, as has been previously demonstrated in yeast [10].

## Materials and methods

### ES cell culture and drug treatment

ES cells used in this study were wild-type OS25 [34], G9a knock out (KO) clone 2-3, G9a wild-type clone col4 (G9a WT), G9a transgene rescue clone 15-3 (G9a tg) [18], Suv39 h1/h2 double KO (DKO) clones DN57/DN72 and wild-type littermate clone wt26 (Suv39 h1/h2 WT) [35], Eed KO clones B1.3/G8.1 [4], Dnmt1 c/c (Dnmt1 KO) [36], Dnmt3a/Dnmt3b DKO clone 10 (Dnmt3a/3b DKO) [27,37], Mbd3 KO clone Fix2 [38], Dicer KO clones D3-S5/D3-S6 and Dicer wild-type clone D3 (described below). ES cells were derived from Dicer flox/flox blastocysts [39] and transfected with the CRE-ER transgene to produce the Dicer flox/flox ES clone D3 (Dicer WT). The Dicer KO clones (D3-S5, D3-S6) were established after tamoxifen treatment (800 nM; Sigma, Poole, UK) of the D3 clone. Deletion of both alleles was confirmed by genotyping.

The ES cell lines were maintained in the undifferentiated state by culturing on gelatinized plates in KO-DMEM (Invitrogen, Carlsbad, CA, USA) supplemented with leukemia inhibitory factor (LIF), 10% ES-tested fetal calf serum (GlobePharm, Surrey, UK), L-glutamine, 2-mercaptoethanol, non-essential amino acids and antibiotics. For Eed KO and Dicer cells (WT and KO), a feeder layer of mitotic inactivated fibroblasts was used and the medium was additionally supplemented with 5% knockout serum replacement (KSR, Invitrogen). OS25 cells were grown on gelatinized plates in Glasgow-MEM (Invitrogen) supplemented with LIF, FCS-gold (PAA, Yeovil, UK), L-glutamine, 2-mercaptoethanol, non-essential amino acids, sodium pyruvate, sodium bicarbonate and antibiotics. All ES cell lines examined in this study were Oct4 positive as determined by immunofluorescence (>92 %, data not shown). Undifferentiated ES cells (OS25) were treated with 10 nM (Sigma) for 48 h, 20 nM TSA for 24 h or 15 μM 5-azacytidine (Sigma) for 72 h.

### Replication timing assay

The protocol described by Azuara *et al*. [6] was used. Briefly, asynchronous cell populations were pulse labeled with bromodeoxyuridine (BrdU; 30 minutes), fixed in 70% ethanol, stained with propidium iodide and fractionated according to DNA content by fluorescence assisted cell sorting (FACS). For ES cells grown on a feeder layer, the feeder cells were removed by differential attachment; less than 1% fibroblasts remained after 20-25 minutes plating in non-gelatinized plates. Pre-plating of feeder-dependent ES cells in this way may result in a slight delay in the apparent time of replication for genes that normally replicate very early in S-phase. Six cell cycle fractions were collected, G1-S, G2 and four fractions covering S-phase, S1-S4, where S1 corresponds to early S-phase and S4 to late S-phase. An equal amount of BrdU labeled *Drosophila *DNA was added to each fraction to control for equal recovery. After isolation of total genomic DNA, the DNA was sheared by sonication, denatured and newly replicated, BrdU-labeled DNA was immunoprecipitated using anti-BrdU antibody (BD, Franklin Lakes, NJ, USA). After purification, quantitative real-time PCR (qPCR) was employed to determine the relative quantity of specific loci in each fraction. The sequences of primers for qPCR analysis are given in Table 3. Locus replication was categorized based on the peak fraction(s) as early (peak in G1 or S1), middle-early (peak in S2), middle (peak in S2 and S3), middle-late (peak in S3) or late (peak in S4 or G2).

**Table 3:** Primer sequences

Locus	Forward	Reverse	T_ann_
**Replication timing/ChIP**			
*Gbe*	GGTGCAGATCATCCCCTTGA	TTACCCGACGGCGAAAG	60
α-*Globin*	CCACAAGCTGCGTGTGGAT	ATGCCGCCTGCCAGGT	60
*Oct4*	GGGTGAGAAGGCGAAGTCTGAA	GTGAGCCGTCTTTCCACCAGG	55
*Nanog*	CCCTCTGAGTTTGACCGGTGA	CAAGCTAGGATGTTAGGTCTCCCTG	60
*Esg1*	AAAGACGAACACAGAGTCAAACACC	CACCTGCTCGATGTGAGACATTC	60
*Zfp57*	TGCAAGATAAGAACGAGGAGCAGGAG	CCTTTGCGGCTTTGTGGATTTGTG	60
*Rex1*	TTTGCGGGAATCCAGCAGT	CGTCCCATCGCCACTCTAGAC	55
*Sox2*	CCATCCACCCTTATGTATCCAAG	CGAAGGAAGTGGGTAAACAGCAC	55
*Nkx2.9*	AAGTGCGAGGCGCTCG	TGGCACCTTCCGGACTTG	60
*Sox3*	TGCCCAGATGGCTTCCTATT	ACCCGGACATTCTCCGCT	60
*Mage a2*	TTGGTGGACAGGGAAGCTAGGGGA	CGCTCCAGAACAAAATGGCGCAGA	60
*Ebf*	AGATCTGGTTGAAGCCCTGTATGG	CATGTCACATCTCAGATCCTGTGTTCT	60
*Mash1*	CCAGGCTGGAGCAAGGGA	CGGTTGGCTTCGGGAGC	55
*β-Globin*	GGTGAACTTTACTGCTGAGGAAAAG	TCACCACCAACCTCTTCAACAT	60
*Myf5*	GGAGATCCGTGCGTTAAGAATCC	CGGTAGCAAGACATTAAAGTTCCGTA	55
*Math1*	CCCTCACTCAGGTCGCCTG	CGTGCGAGGAGCCAATCA	55
*Sox1*	ACAAGAGGAGGCAGCGAACC	TCGCAGGTGGAAAGTTTCTCC	55
*Msx1*	ACAGAAAGAAATAGCACAGACCATAAGA	TTCTACCAAGTTCCAGAGGGACTTT	55
*Frg1*	AAGGAGCCTATATCCATGCACTGGAC	GCCTCCCTGCCATTGCTTGT	60
*Slc7a2*	GACAAGGAACAGGGCGAGAAG	CTTTCCTCATCCTGGGCTTGAGTA	60
*Msr1*	GCCACCAATGCCCTAGAATTTC	GGCAGGCTCTCACTAGGAAGC	60
*Loc2*	ACTAGCAACTGGACATAAGAGTACACTACC	ATTACATATGGTGTCTGGAAGCCAG	60
*Adam 26*	CCTTGAACAACGCCCTTTTGTG	GCAAGCTCCCAAAACAGGTGT	60
*Loc4*	TAAGGTAGGCAGTGAGAGACATCCA	GGTGTAAGAAGGTTAGAACTAA	60
X141	GGGTCATAAAACGCTTTTCCAGGAA	TAGCACTGGAGATCAGATTGACGCCT	60
Minor satellite	TGATATACACTGTTCTACAAATCCCGTTTC	ATCAATGAGTTACAATGAGAAACATGGAAA	55
Major satellite	GACGACTTGAAAAATGACGAAATC	CATATTCCAGGTCCTTCAGTGTGC	55
rDNA	CCTGTGAATTCTCTGAACTC	CCTAAACTGCTGACAGGGTG	60
IAP	TTGATAGTTGTGTTTTAAGTGGTAAATAAA	AAAACACCACAAACCAAAATCTTCTAC	60
LINE 1	TTTGGGACACAATGAAAGCA	CTGCCGTCTACTCCTCTTGG	60
SINE B1	GTGGCGCACGCCTTTAATC	GACAGGGTTTCTCTGTGTAG	60
**Gene expression**			
*Mage a2*	GAAGATCTCAGGAGTGTCAGGACTG	TCAGCCATTATGACTGTCCTAGGTAA	60
*Ubc*	AGGAGGCTGATGAAGAGCTTGA	TGGTTTGAATGGATACTCTGCTGGA	60
*Oct4*	CCCAAGGTGATCCTCTTCTGCTT	GAGAAGGTGGAACCAACTCCCG	60
**CpG methylation assay**			
*Major sat*	GACGACTTGAAAAATGACGAAATC	CATATTCCAGGTCCTTCAGTGTGC	55
*Sox2*	TGGACTGCGAACTGGAGAAGG	CGCCCGGAGTCTAGCTCTAAATATT	60

IAP, intracisternal A particle. T_ann_, annealing temperature.

#### Note regarding replication timing of repeated sequences

As mentioned above, we assessed the proportion of a specific DNA sequence within newly replicated DNA for each cell cycle fraction relative to the total from all six fractions. This means that for single copy genes, a change in one allele will give a shift for 50% of the signal whereas for a multi-copy locus, only a small fraction (1% for a sequence repeated 100 times) will shift. Changes in single copy loci are, therefore, much more readily detected than changes in repeated sequences. Variability in locus replication among multi-copy loci would be predicted to result in a spread-out signal detected across multiple cell cycle fractions.

### ChIP

Exponentially growing wild-type ES cells (OS25) were paraformaldehyde (1%) fixed for 10 minutes at room temperature, lysed and chromatin immuno-precipitated essentially as described [40]. Briefly, chromatin (50 μg) was pre-cleared 2 h at 4°C, incubated with antibodies (2 μl IgG (Z0259, DAKO, Copenhagen, Denmark); 2 μl anti-H3 (ab-1791-100, binds H3 independent of modification state, Abcam, Cambridge, UK), 5 μl anti-H3K9me2, 10 μl anti-H3K9me3 or 5 μl anti-H3K9ac (07-441, 07-442, 07-352, Upstate/Millipore, Billerica, MA, USA) at 4°C over night (ON) and the immune-complexes collected by adding protein-A sepharose (Sigma) (incubated 2 h at 4°C). Unbound chromatin was removed by washing 4× in ChIP wash buffer (0.1% SDS, 1% Triton X-100, 2 mM EDTA, 150 mM NaCl, 20 mM Tris.Cl pH 8.1 and protease inhibitors) and 1× in high salt ChIP wash buffer (0.1% SDS, 1% Triton X-100, 2 mM EDTA, 150 mM NaCl, 20 mM Tris.Cl pH 8.1 and protease inhibitors) after which 250 μl elution buffer was added (1% SDS, 0.1 M NaHCO_3_, 100 μg/ml RNaseA, 500 μg/ml Proteinase K). After incubating at 37°C for 2 h and at 65°C ON, DNA was purified using a Gel purification kit (Qiagen, Crawley, UK), using 2 × 40 μl of 10 mM Tris.Cl pH 8 for elution. ChIP samples were analyzed by qPCR (sequences of primers are given in Table 3) or microarray hybridization.

### Microarray analysis

Input and ChIP samples were amplified by LM-PCR as advised by Nimblegen, Reykjavik, Iceland. Labeling and hybridization was done by Nimblegen using a custom designed 50mer tiling array (100 bp average resolution) covering a region from 100 kb upstream to 100 kb downstream of the analyzed genes. Normalized and scaled Chip: input ratios for anti-H3K9ac and anti-H3 ChIP hybridizations were produced by Nimblegen. The log2(H3K9ac/H3) ratios were calculated from these data and plotted against the chromosomal position of the probes. Blue lines in Figure 2 indicate peaks in the dataset detected by a hidden Markov model-based algorithm using TileMap [41]. Gaps in the profile arise from repetitive regions in the genome that are not represented on the array.

### Analysis of transcript levels

RNA was isolated from 3-5 × 10^6 ^cells using the RNAeasy mini prep kit (Qiagen) with on-column DNase treatment. To remove residual genomic DNA, RNA (1.2 μg) was treated with RNAfree (Ambion, Austin, TX, USA) for 50 minutes before reverse transcription using SuperScript III (Invitrogen) and random primers in 20 μl reactions according to the manufacturer's instructions.

### qPCR analysis

The sequence of primer pairs used in this study is given in Table 3. All primer pairs were tested for efficiency (>1.95) and linearity (R^2 ^> 0.99). Reactions (30 μl) were set up using a Qiagen SYBR green kit with the appropriate template (2 μl (corresponding to 200 cell equivalents) for replication timing, 1.5 μl of 1:5 diluted cDNA for gene expression, 2% of eluted DNA for ChIP, 2 μl (corresponding to 1.7 ng) genomic DNA for analysis of DNA methylation) and analyzed on Chromo4™ Real-Time PCR Detector (Bio-Rad, Hercules, CA, USA) with Opticon Monitor™ software.

### DNA methylation assay

Triplicate reactions of genomic DNA with or without the methylation sensitive restriction enzyme HpyCh4IV (New England Biolabs, Beverly, USA) were incubated for 3 h and the extent of digestion analyzed by qPCR. The primers for the major satellite span a HpyCh4IV site. The Sox2 primers, which do not span a HpyCh4IV site, were used to control for equal DNA content.

## Additional data files

The following additional data are available with the online version of this paper. Additional data file 1 contains supplementary materials and methods, legends to supplementary Figures 1-4, and supplementary Tables 1 and 2. Supplementary Table 1 contains the percentages of cells positive for ES cell markers and Supplementary Table 2 contains coordinates, GC content and Line density of the genes analysed in Figure 1b. Additional data file 2 contains supplementary Figures 1-4. Supplementary Figure 1 shows alkaline phosphatase staining and cell cycle profiles of the mutant ES cell lines analysed. Supplementary Figure 2 contains replication timing profiles of all loci analysed in each of the mutant ES cell lines analysed. Supplementary Figure 3 shows analysis of the *Mage a2 *gene. Supplementary Figure 4 shows analysis of short hairpin RNA mediated knockdown of Eed in Dnmt1 mutant ES cells. Additional data file 3 is an archive containing microarray data for the histone acetylation analysis.

## Abbreviations

BrdU, bromodeoxyuridine; ChIP, chromatin immunoprecipitation; DKO, double knock out; Dnmt, DNA methyl transferase; ES, embryonic stem; HDAC, histone deacetylase; HMTase, histone methyl transferase; KO, knock out; LINE, long interspersed nuclear element; PRC, polycomb repressor complex; qPCR, quantitative real-time PCR; SINE, short interspersed nuclear element; siRNA, short interfering RNA; TSA, trichostatin A.

## Authors' contributions

HFJ, VA and AGF designed the study described in this report. HFJ performed the experiments, analysed the data, and was responsible for writing the initial versions of the manuscript. SA performed cell culture experiments. MS and AT were involved in performing the microarray analysis. MS analysed the microarray data. TN, BSC and BR contributed material/reagents/analysis tools. MM and AGF supervised and oversaw the completion of the studies as well as the writing of the manuscript. All authors read and approved the final version of the manuscript.

## Supplementary Material

Additional data file 1 Supplementary Table 1 contains the percentages of cells positive for ES cell markers and supplementary Table 2 contains coordinates, GC content and line density of the genes analyzed in Figure 1b. Click here for file

Additional data file 2 Supplementary Figure 1 shows alkaline phosphatase staining and cell cycle profiles of the mutant ES cell lines analyzed. Supplementary Figure 2 contains replication timing individual profiles of all genes in each of the mutant ES cell lines analyzed. Supplementary Figure 3 shows analysis of the *Mage a2 *gene. Supplementary Figure 4 shows analysis of short hairpin RNA mediated knockdown of Eed in Dnmt1 mutant ES cells. Click here for file

Additional data file 3 Microarray data for the histone acetylation analysis. Click here for file
