# Supplementary material for: The impact of chromatin modifiers on the timing of locus replication in mouse embryonic stem cells
Source: Genome Biol. 2007 Aug 17;8(8):R169. doi: 10.1186/gb-2007-8-8-r169 (PMC2374999; doi:10.1186/gb-2007-8-8-r169)
Supplement: Additional data file 1 — Supplementary Table 1 contains the percentages of cells positive for ES cell markers and supplementary Table 2 contains coordinates, GC content and line density of the genes analyzed in Figure 1b. [file gb-2007-8-8-r169-S1.pdf]

Supplementary information found in additional data files 1-3

Supplementary Materials and methods (additional data file 1)
Legend to Supplementary Figures (additional data file 1)
Supplementary Table 1 (additional data file 1)
Supplementary Table 2 (additional data file 1)
Supplementary Figure 1 (additional data file 2)
Supplementary Figure 2 (additional data file 2)
Supplementary Figure 3 (additional data file 2)
Supplementary Figure 4 (additional data file 2)
Supplementary Archive (additional data file 3)

Supplementary Materials and methods

Oct4 staining: Cells were harvested by trypsinisation, washed in PBS and fixed in 0.1% paraformaldehyde in PBS for 10 min at 37 ˚C. Following fixation the cells were washed in PBS and permeabilised with cold 90% Methanol for 30 min on ice. Methanol was removed by washing 2x in PBS with 1% BSA and 0.1% Triton X-100 and cells (1-5 x10^5) were incubated with anti-Oct4 antibody (Santa Cruz N-19, 1:100) for 1h at room temperature. Cells were washed 2x in PBS with 1% BSA and 0.1% Triton X-100 before incubating with an Alexa-568 coupled anti-Goat antibody (Molecular Probes) for 30 min at room temperature. Before analysis on a FACScalibur the cells were washed 2x in PBS with 1% BSA and 0.1% Triton. The profile of Oct4 stained cells was compared to cells stained with the secondary antibody only.

SSEA-1 staining: Cells were harvested by trypsinisation using 0.025% Trypsin + 1% chicken serum and washed 3x in PBS with 1% BSA. Cells (1-5 x10^5) were resuspended in 35 ul of APC-coupled anti-SSEA-1 (R&D, 1:3.5). Following 30-45 incubation at 4 ˚C, the cells were washed 2x in PBS with 1% BSA and analysed on a FACScalibur. SSEA-1 stained cells were compared to cells stained with APC-coupled non-relevant antibody (anti-B220).

Analysis of alkaline phosphatase activity: Cells (1-5,000) were seeded in a 35 mm dish and cultured for 3-5 days before fixation and staining using a kit for measuring alkaline phosphatase activity using the manufacturers instructions (procedure 86, Sigma). The stained colonies were analysed using a Leica microscope with a 10x objective.

Short hairpin RNA mediated knock down of Eed: Dnmt1 KO ES cells were transfected with a pSuper(Neo-IRES-GFP)-based shRNA construct targeting Eed (target sequence 1: GAAGCAACAGAGTAACCTT, target sequence 2: GCCTCAAGGAAGATCATAA) or control sequence (control sequence: GCGCGCTTTGTAGGATTCG) using Lipofectamine (Invitrogen) as described by the manufacturer. Three days after transfection, the cells were harvested by

trypsinisation and FACS sorted for GFP expression. Sorted cell population were analysed for purity (>95%) before fixation and processing for replication timing analysis or analysis by western blotting to confirm efficient knockdown of Eed.

Legend to Supplementary Figures

Legend to Supplementary Figure 1. (**a**) Wild type and mutant ES cells lines analysed here all have alkaline phosphatase activity. Single colonies cultured for 3-5 days were analysed. (**b**) Cell cycle profiles of all wild type and mutant mES cells analysed. Cells were fixed in 70% EtOH, stained with propidium iodide and analysed by FACS.

Legend to Supplementary Figure 2. Individual replication timing profiles for all genes summarised in figure 1 and 3 in each of four wild type ES cell lines, OS25, G9a WT, Suv39 h1/h2 WT, Dicer WT (white bars) and eight mutant ES cell lines Mll KO, Eed KO, Dnmt 1 KO, Dnmt 3a/3b DKO, Mbd3 KO, G9a KO, Suv39 h1/h2 DKO and Dicer KO (grey bars). These replication profiles are averages of 2-5 experiments, except for certain wild type profiles, where, due to the consistent profiles in wild type cells, some profiles represent a single experiment. Error bars show standard deviations.

Legend to Supplementary Figure 3. *Mage a2* is a G9a target in ES cells [57] and its expression has been shown to be sensitive to DNA methylation [19]. A gene expression analysis confirmed the upregulation of *Mage a2* in G9a null ES cells (**a**), and also revealed low expression in Dnmt 1 KO and Dnmt 3a/3b DKO cells (data not shown). The *Mage a2* promoter was found to be di-methylated at H3K9 in wild type ES cells (**b**), consistent with the enzymatic activity of G9a (Supplementary Table 1). Enrichment of dimethyl-H3K9 at the promoters of *Oct4*, *Hox A7*, *Mage a2* and at the major satellite in wild type ES cells (OS25) was measured by ChIP analysis. The enrichment relative to H3 is shown. In wild type ES cells as well as in other mutant lines, the *Mage a2* locus replicates mid-late but this is advanced to mid-early in G9a KO cells (C and Figure 1**a**). Importantly, transgenic rescue of the G9a null cells reverts *Mage a2* replication timing to mid-late in S-phase (**c**, bars show the relative

locus quantity in each fraction). The transgenic rescue also re-establishes silencing of this locus (**a**).

Legend to Supplementary Figure 4. (**a**) Transfection of Dnmt 1 KO ES cells with Eed-targeting shRNA constructs results in reduced Eed protein levels as shown by western blotting. Equal loading is demonstrated by the anti-Lamin blot. (**b**) The replication timing profiles of single copy genes (*Oct4*, *Rex1*, *Nkx2.9*, *Ebf*, *Mash1*, *Sox3* and *β-Globin*) and repeat sequences (Major and minor satellite) is not affected beyond changes observed in the Dnmt 1 KO and Eed KO mutant ES cells lines. Histograms show the average relative locus replication within each cell cycle fraction for two independent experiments. White bars represent Dnmt 1 KO ES cells transfected with a control shRNA construct whereas black and gray bars show the profile of Dnmt 1 KO ES cells transfected with Eed targeting shRNA constructs (black bars: sequence 1 + 2, grey bars: sequence 1 only).

Supplementary Table 1

Cell line	Oct4 positive (%)	SSEA-1 positive (%)	SSEA-1 high (%)
WT OS25	97	99	89
Mll KO	88	97	89
Eed KO (B1.3)	71	85	64
Eed KO (G8.1)	90	93	79
Dnmt1 KO	98	99	99
Dnmt3a/3b DKO	99	98	98
Mbd3 KO	95	98	73
G9a WT	90	97	95
G9a KO	96	97	94
Suv 39h1/h2 WT	99	98	94
Suv 39h1/h2 DKO (DN57)	99	96	95
Suv 39h1/h2 DKO (DN72)	98	95	92
Dicer WT	96	96	89
Dicer KO (D3-S5)	94	91	75
Dicer KO (D3-S6)	80	93	76
Primary Embryonic Fibroblast	<1	<1	<1

Supplementary Table 2

Gene name	Localisation (Assembly: Mouse May 2004)	GC content %	Line density %
Nanog	chr6:123,245,165-123,452,206	44.8	4.52
Zfp57	chr17:35,425,987-35,633,584	44.4	5.78
Oct4	chr17:33,914,518-34,121,673	48.1	2.51
Esg1	chr9:78,663,904-78,864,145	44.2	3.81
Sox2	chr3:34,352,008-34,556,333	44.4	3.14
Rex1	chr8:42,312,560-42,526,503	41.3	13.66
Nkx2.9	chr12:51,704,957-51,906,245	42.7	15.15
Mage a2	chrX:144,842,761-145,048,852	40.9	40.07
Ebf	chr11:44,157,868-44,744,905	42.1	6.30
Mash1	chr10:87,138,517-87,340,872	42.0	16.03
Sox3	chrX:52,357,714-52,559,774	41.2	17.09
β-Globin	chr7:91,270,307-91,471,721	38.4	27.61
NeuroD1	chr2:79,246,699-79,450,695	40.1	14.25
Myf5	chr10:107,056,891-107,260,118	38.4	14.00
