# Supplementary material for: The impact of chromatin modifiers on the timing of locus replication in mouse embryonic stem cells
Source: Genome Biol. 2007 Aug 17;8(8):R169. doi: 10.1186/gb-2007-8-8-r169 (PMC2374999; doi:10.1186/gb-2007-8-8-r169)

Supplementary Figure 1a

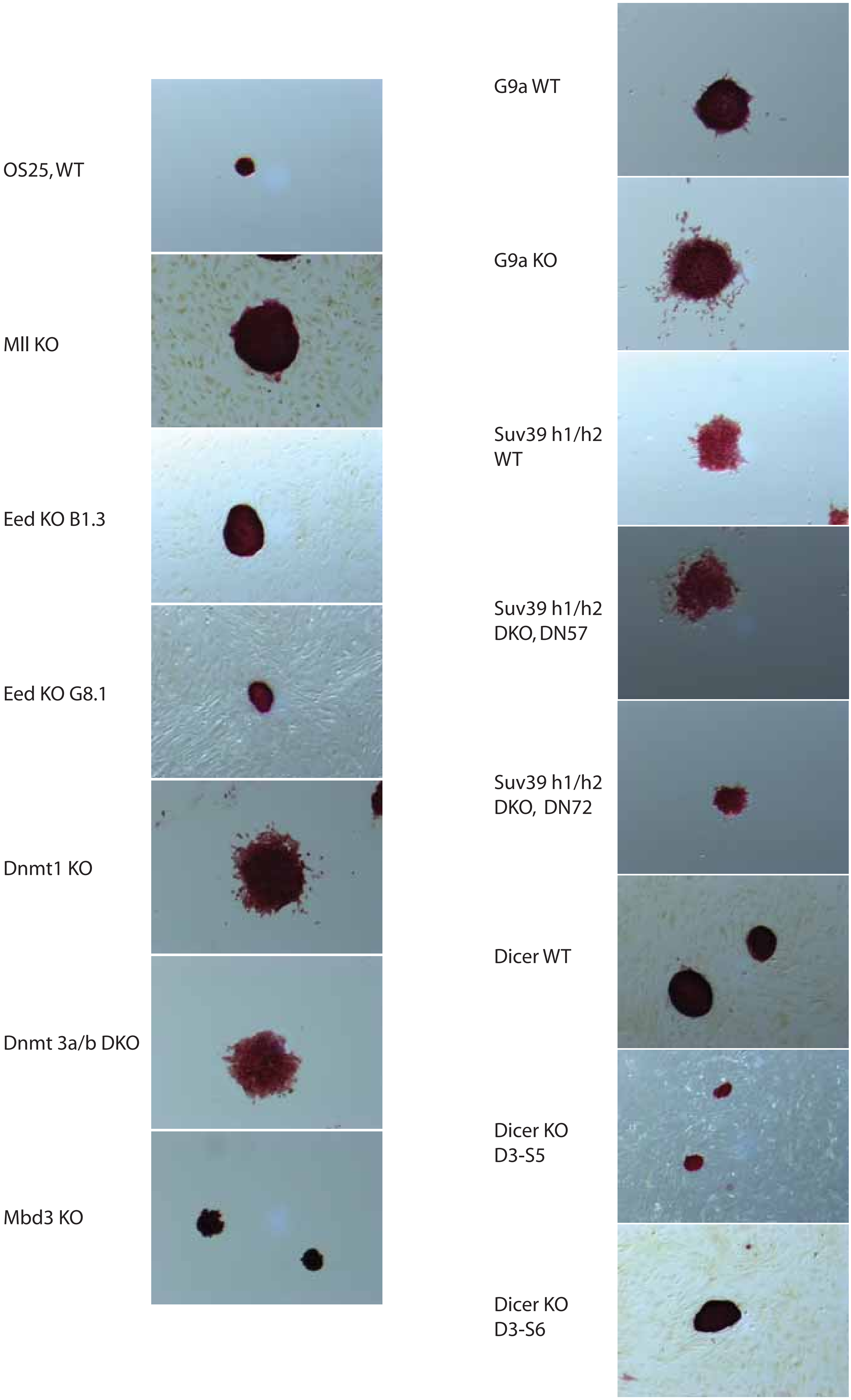

Supplementary Figure 1b

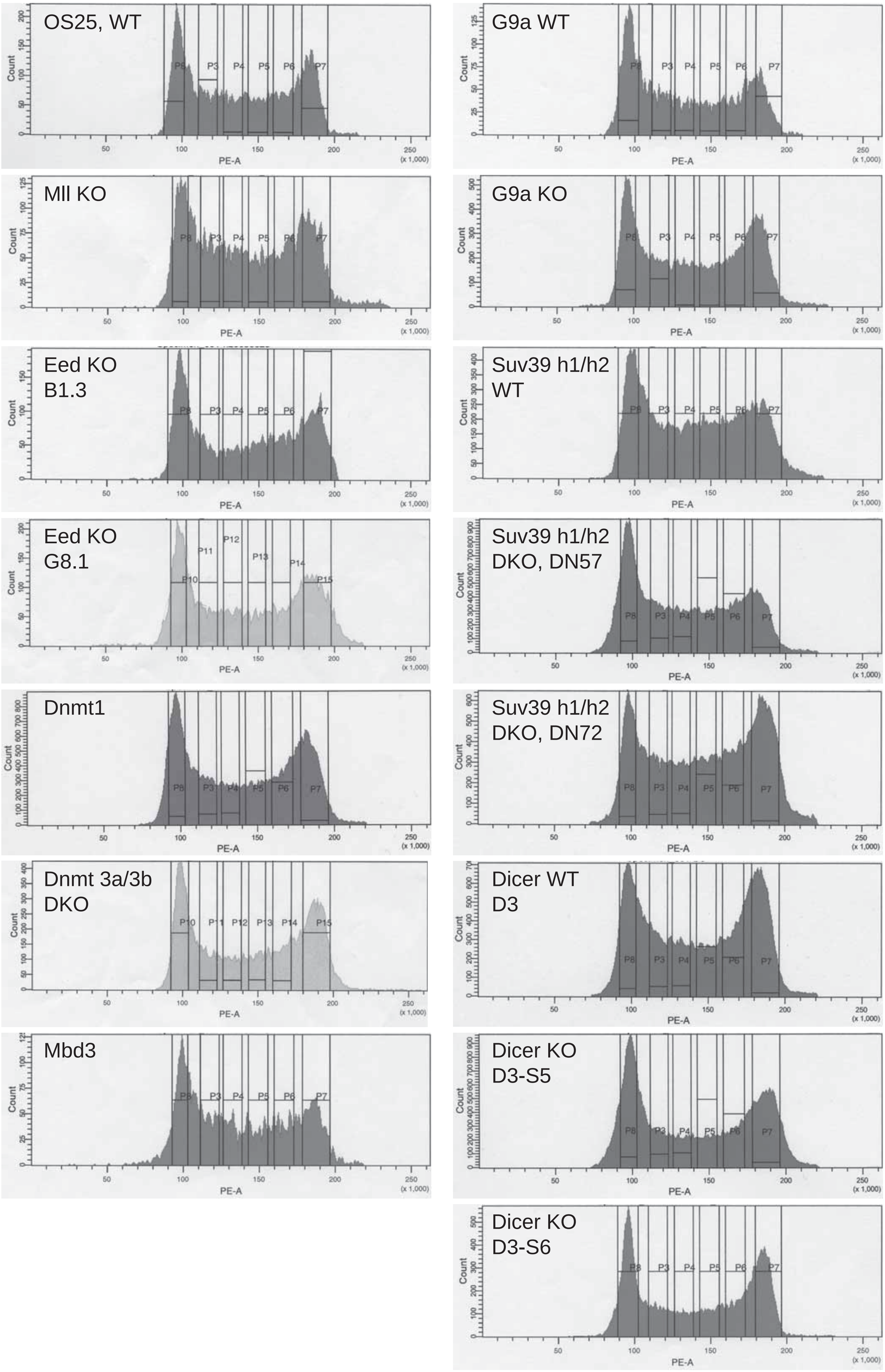

Supplementary Figure 2 p.1/3

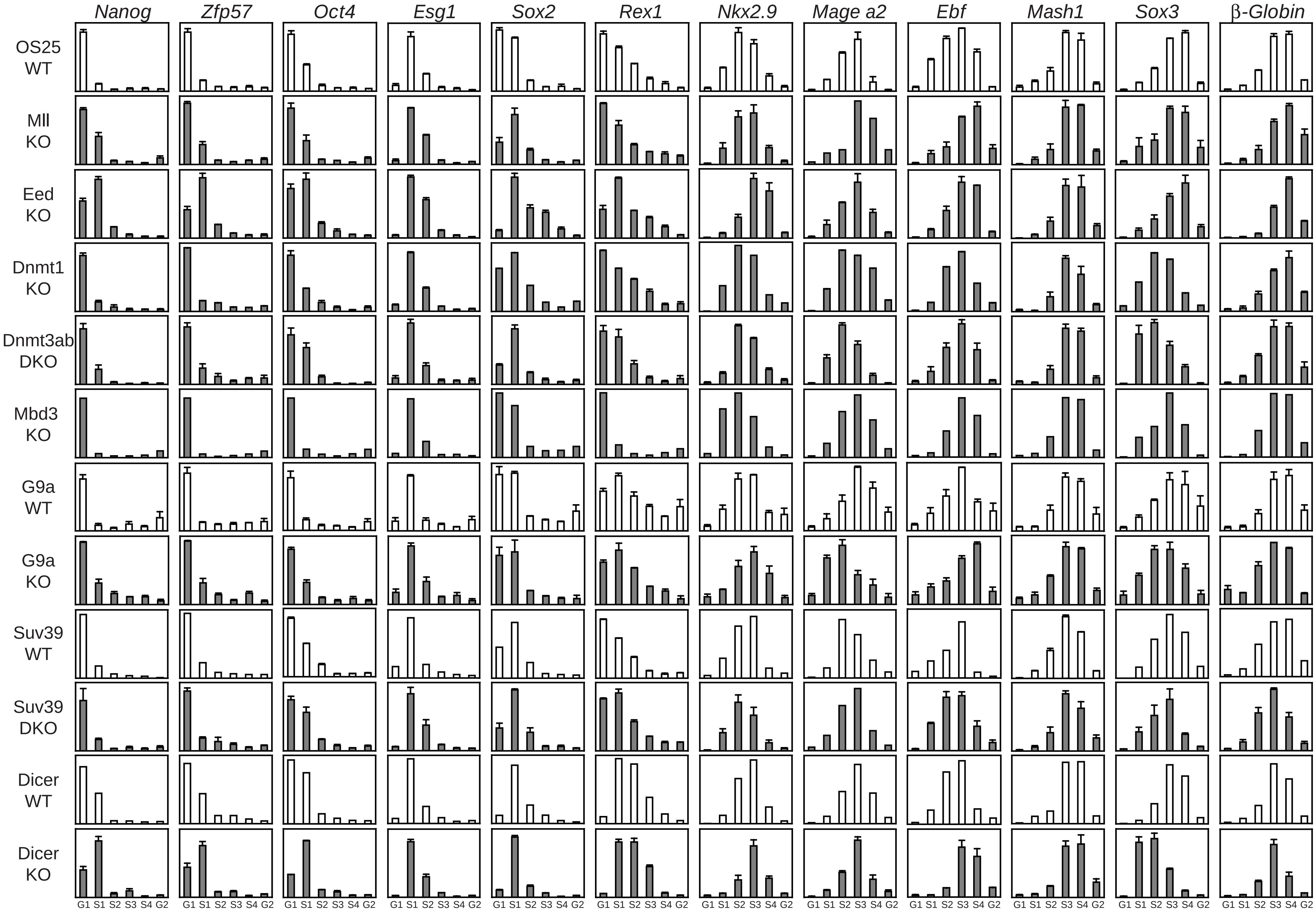

Supplementary Figure 2 p.2/3

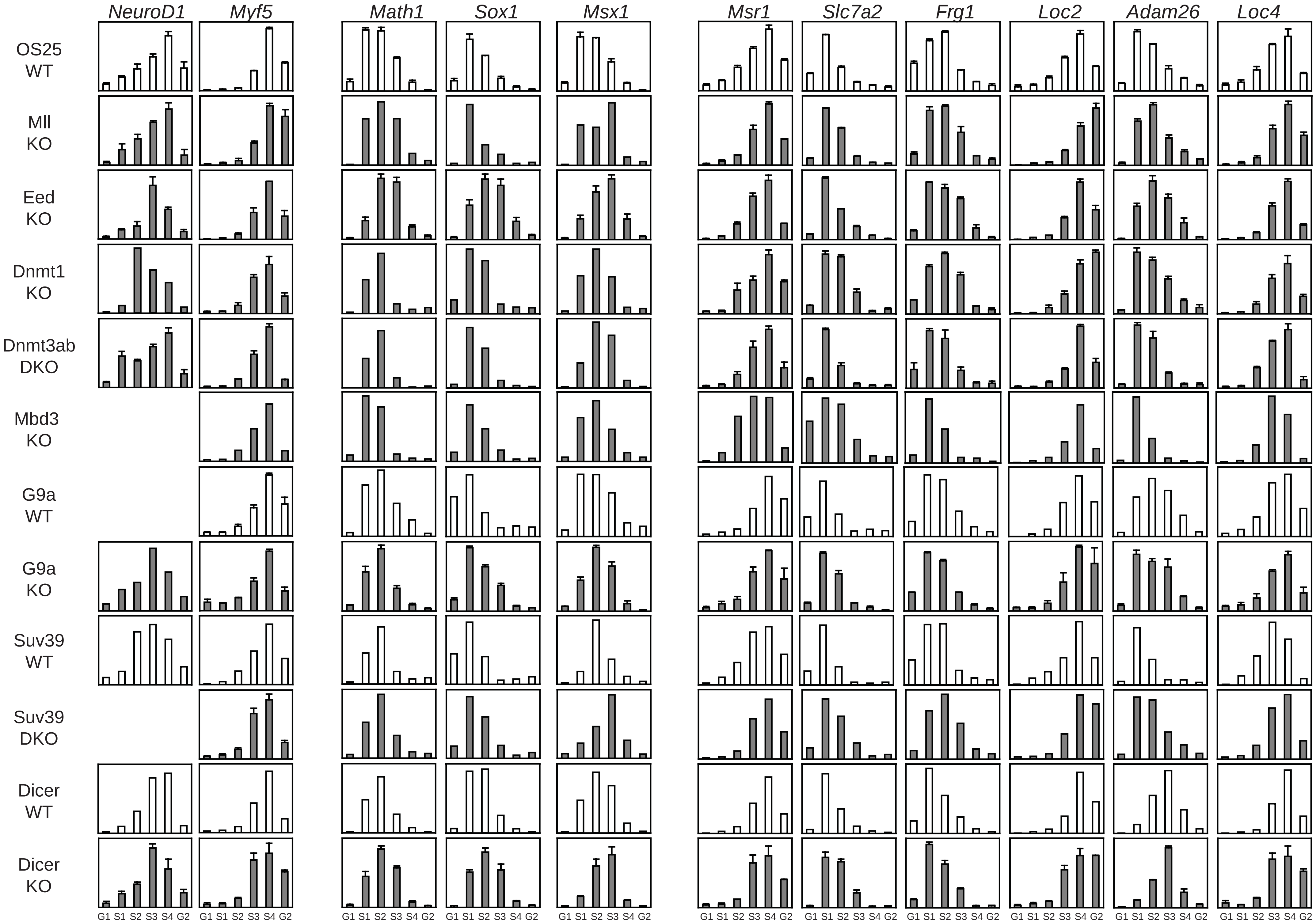

Supplementary Figure 2 p.3/3

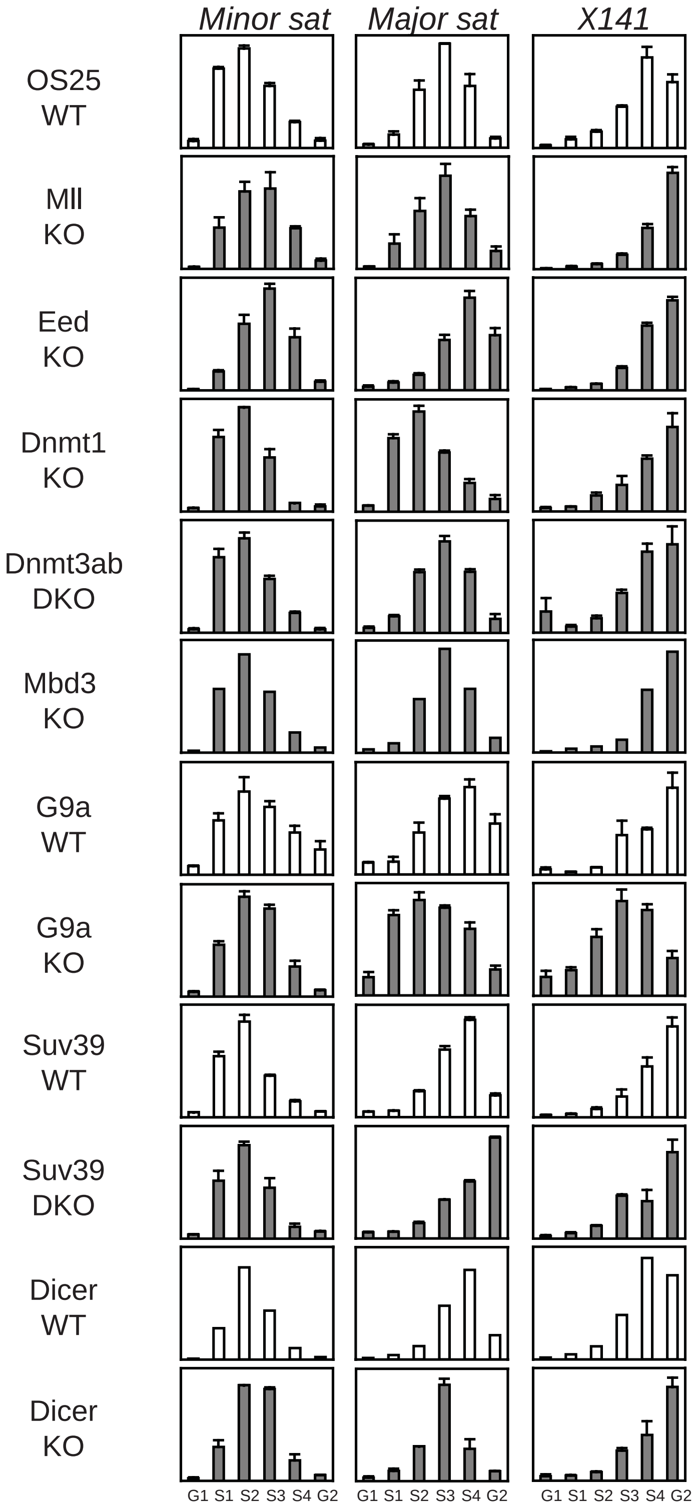

Supplementary Figure 3

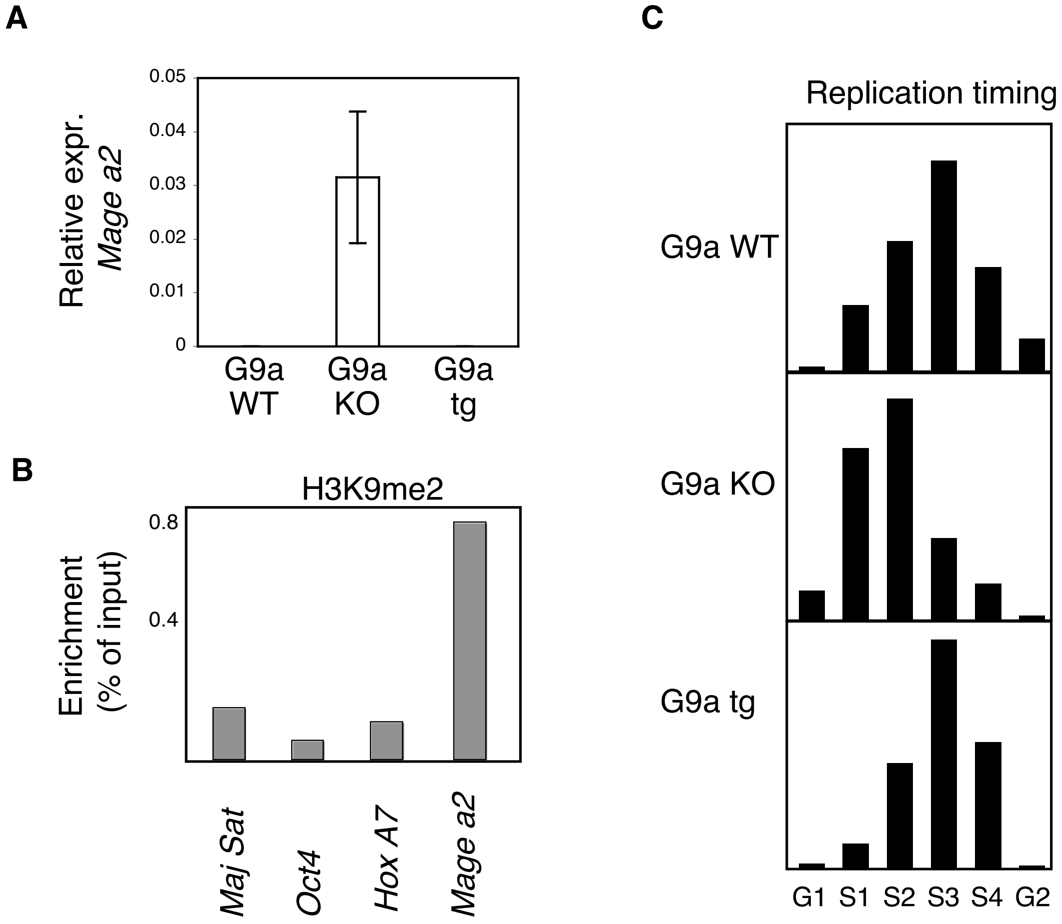

Supplementary Figure 4

a

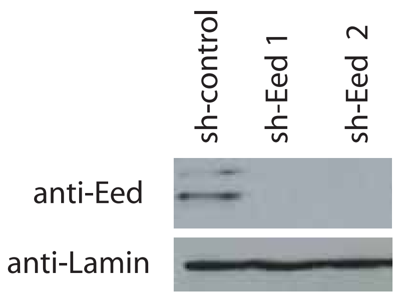

b

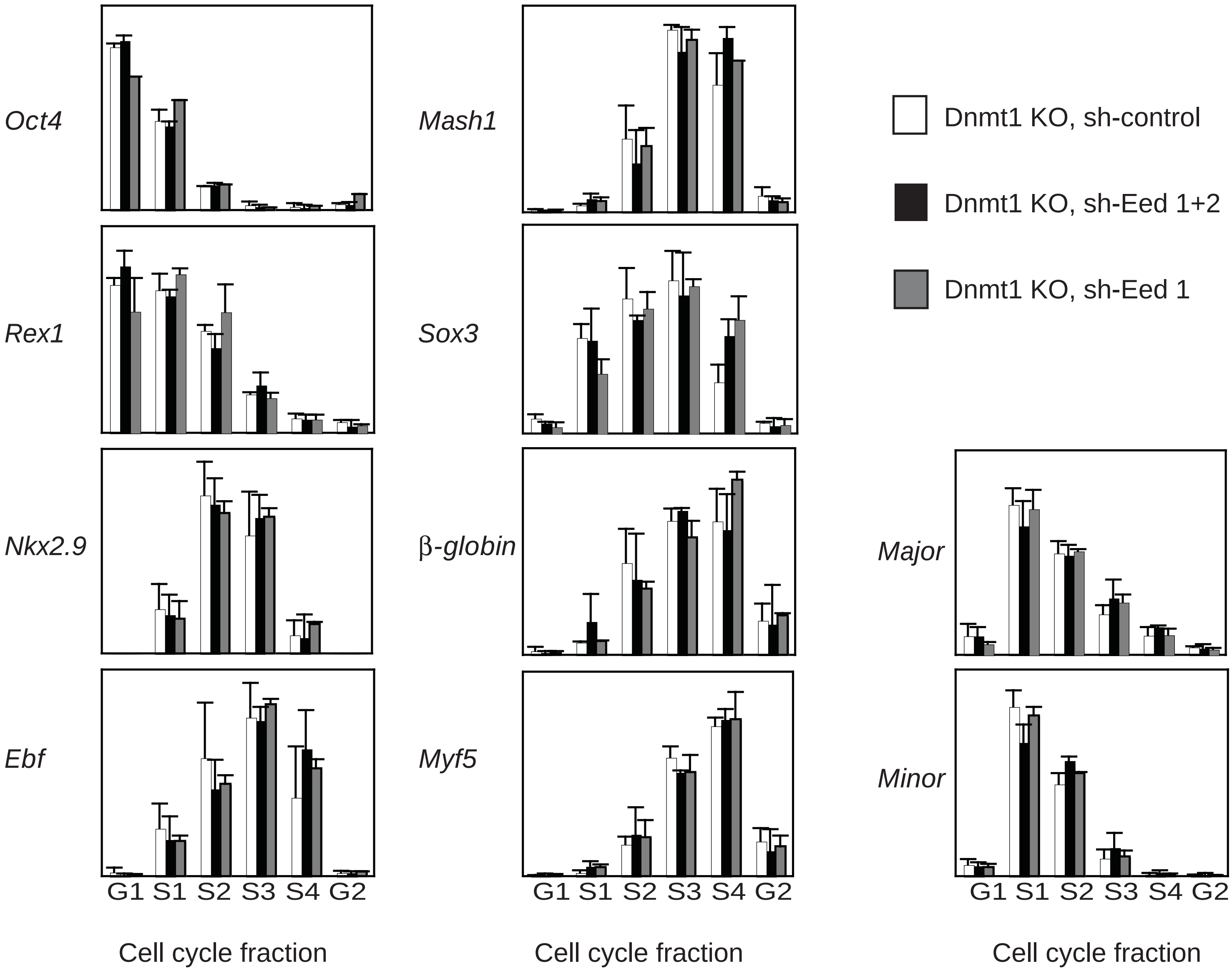

Supplement: Additional data file 2 — Supplementary Figure 1 shows alkaline phosphatase staining and cell cycle profiles of the mutant ES cell lines analyzed. Supplementary Figure 2 contains replication timing individual profiles of all genes in each of the mutant ES cell lines analyzed. Supplementary Figure 3 shows analysis of the Mage a2 gene. Supplementary Figure 4 shows analysis of short hairpin RNA mediated knockdown of Eed in Dnmt1 mutant ES cells. [file gb-2007-8-8-r169-S2.pdf]
